# Ling-Gui-Zhu-Gan decoction ameliorates nonalcoholic fatty liver disease via modulating the gut microbiota

**DOI:** 10.1128/spectrum.01979-23

**Published:** 2024-04-22

**Authors:** Lu-ping Chen, Lin-fang Zhang, Shuang Liu, Hua Hua, Lei Zhang, Bao-cheng Liu, Rui-rui Wang

**Affiliations:** 1 Shanghai Innovation Center of TCM Health Service, Shanghai University of Traditional Chinese Medicine, Shanghai, China; 2 Oxford Suzhou Centre for Advanced Research, Suzhou Industrial Park, Jiangsu, China; 3 Shanxi Institute for Function Food, Shanxi Agricultural University, Taiyuan, Shanxi, China; 4 Sichuan Institute for Translational Chinese Medicine, Chengdu, China; 5 Sichuan Academy of Chinese Medical Sciences, Chengdu, China; Wayne State University, Detroit, Michigan, USA

**Keywords:** Ling-Gui-Zhu-Gan decoction, nonalcoholic fatty liver disease, gut microbiota, bile acid, short-chain fatty acids

## Abstract

Numerous studies have supported that nonalcoholic fatty liver disease (NAFLD) is highly associated with gut microbiota dysbiosis. Ling-Gui-Zhu-Gan decoction (LG) has been clinically used to treat NAFLD, but the underlying mechanism remains unknown. This study investigated the therapeutic effect and mechanisms of LG in mice with NAFLD induced by a high-fat diet (HD). An HD-induced NAFLD mice model was established to evaluate the efficacy of LG followed by biochemical and histopathological analysis. Metagenomics, metabolomics, and transcriptomics were used to explore the structure and metabolism of the gut microbiota. LG significantly improved hepatic function and decreased lipid droplet accumulation in HD-induced NAFLD mice. LG reversed the structure of the gut microbiota that is damaged by HD and improved intestinal barrier function. Meanwhile, the LG group showed a lower total blood bile acids (BAs) concentration, a shifted BAs composition, and a higher fecal short-chain fatty acids (SCFAs) concentration. Furthermore, LG could regulate the hepatic expression of genes associated with the primary BAs biosynthesis pathway and peroxisome proliferator-activated receptor (PPAR) signaling pathway. Our study suggested that LG could ameliorate NAFLD by altering the structure and metabolism of gut microbiota, while BAs and SCFAs are considered possible mediating substances.

**IMPORTANCE:**

Until now, there has still been no study on the gut microbiota and metabolomics of Ling-Gui-Zhu-Gan decoction (LG) in nonalcoholic fatty liver disease (NAFLD) mouse models. Our study is the first to report on the reshaping of the structure and metabolism of the gut microbiota by LG, as well as explore the potential mechanism underlying the improvement of NAFLD. Specifically, our study demonstrates the potential of gut microbial-derived short-chain fatty acids (SCFAs) and blood bile acids (BAs) as mediators of LG therapy for NAFLD in animal models. Based on the results of transcriptomics, we further verified that LG attenuates NAFLD by restoring the metabolic disorder of BAs via the up-regulation of Fgf15/FXR in the ileum and down-regulation of CYP7A1/FXR in the liver. LG also reduces lipogenesis in NAFLD mice by mediating the peroxisome proliferator-activated receptor (PPAR) signaling pathway, which then contributes to reducing hepatic inflammation and improving intestinal barrier function to treat NAFLD.

## INTRODUCTION

Nonalcoholic fatty liver disease (NAFLD) has emerged as one of the leading causes of chronic liver disease globally, with a prevalence of approximately 30% in the general population and 80% among patients who are morbidly obese (1). Meanwhile, NAFLD is considered to increase the risk of a wide range of diseases, including type II diabetes, cardiovascular disease, and even cancer. However, effective treatment approaches for NAFLD remain limited (2). An increasing number of studies have suggested that traditional Chinese medicine (TCM) may have certain advantages in the prevention and treatment of NAFLD (3, 4).

The ancient classical formula Ling-Gui-Zhu-Gan decoction (LG) was first documented in *Shanghan Zabing Lun* thousands of years ago and has been applied in the alleviation of dizziness and palpitations for a long time in China (5). Modern medical studies have found that LG can regulate genes related to lipid metabolism, and reduce the levels of fasting lipids, oxidative stress, and inflammatory cytokines (6–8). The most recent clinical study found LG improves insulin resistance in overweight subjects with NAFLD (9). Despite this, the direct target and effective mechanisms of LG remain unclear due to the complex chemical compositions and low blood concentration of TCM.

Recently, accumulating studies have discovered that the gut microbiota is involved in the pathogenesis of NAFLD (10, 11). It has been reported that germ-free mice transplanted with gut microbiota from high-fat diet (HD) responder donors exhibit NAFLD predispositions (12). The metabolites of gut microbiota, such as bile acids (BAs) and short-chain fatty acids (SCFAs), also participate in the occurrence and development of NAFLD (13). BAs not only play important roles in the digestion of lipids but also function as signaling molecules with a profound effect on metabolism and energy balance (14, 15). SCFAs fermented from complex carbohydrates by bacteria can protect intestinal barrier integrity and maintain immune homeostasis (16). In this regard, the gut microbiota is considered a potential target for preventing and treating NAFLD.

The aim of this study was to investigate the protective role of LG on the development of HD-induced NAFLD by exploring the structure and metabolism of the gut microbiota with analytical methods of metagenomics and metabolomics, which may support new strategies for the management of NAFLD by identifying potential molecular mechanisms of TCM treatment.

## RESULTS

### High-performance liquid chromatography analysis of LG

As part of a standardization process, we carried out a high-performance liquid chromatography (HPLC) fingerprint analysis of LG. In this HPLC fingerprint, LG and the reference standards were compared to identify the major peaks. There are seven compounds in LG, viz. (i) liquiritin apioside; (ii) liquiritin; (iii) isoliquiritin; (iv) cinnamic acid; (v) cinnamal dehyde; (vi) licoricesaponin G2; and (vii) ammonium glycyrrhizinate were properly identified (Fig. S1).

### LG improved the metabolic phenotype of NAFLD mice

**Fig 1:**
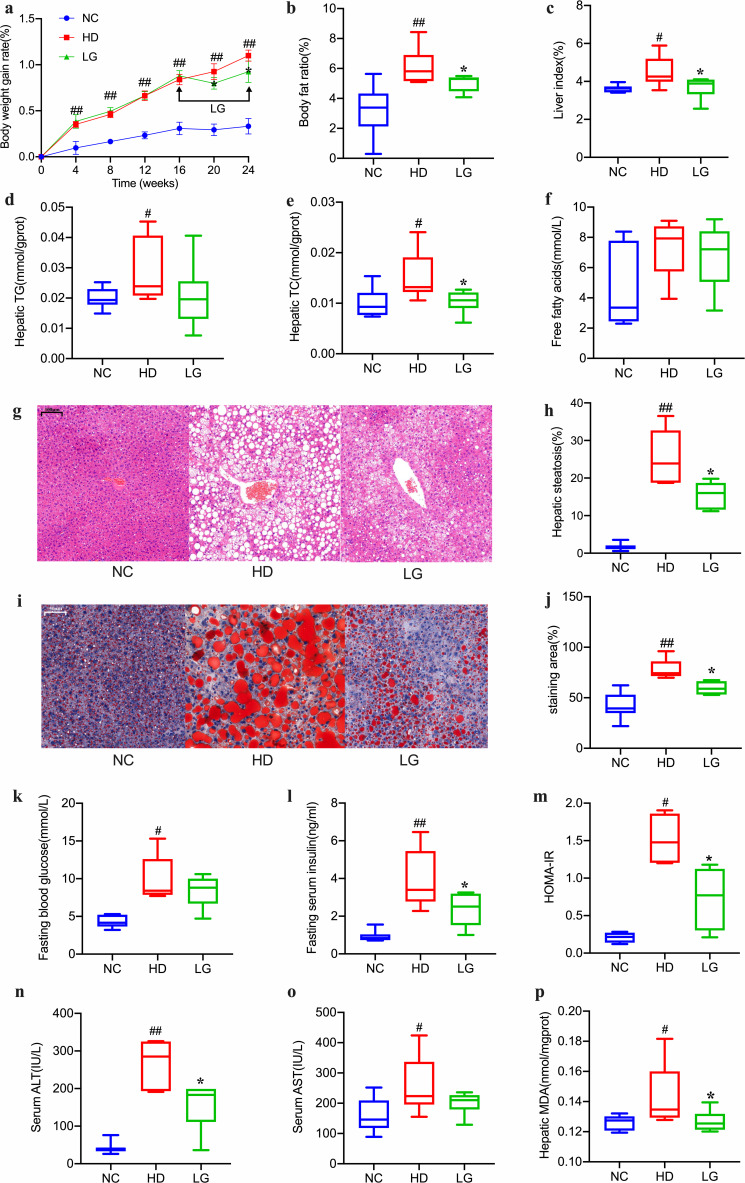
Effects of LG administration on NAFLD mice: (a) body weight gain rate with time (LG treatment started from the 16th week); (b) body fat ratio; (c) liver index; (d) hepatic TG level; (e) hepatic TC level; (f) hepatic free fatty acid level; (g) HE-stained liver section (magnification, ×100); (h) bar graph of the volume density of liver steatosis; (i) oil red O-stained liver section (magnification, ×200); (j) quantitative results of oil red O staining; (k) fasting blood glucose; (l) fasting serum insulin; (m) HOMA-IR; (n) serum ALT level; (o) serum AST level; (p) hepatic MDA level. # *P* < 0.05, ## *P* < 0.01 compared with the NC group. *P < 0.05, **P*P* < 0.01 compared with the HD group. Box plots display medians with interquartile ranges.

Our results showed that the body weight gain rate of the mice was significantly higher in the groups fed with HD than in the group fed with a normal chow diet (NC). After 4 weeks of administration, the body weight gain rate of the LG group was significantly lower than that of the HD group (Fig. 1a). The body fat ratio and liver index of the HD group were significantly higher than those of the NC group, which decreased significantly with LG treatment (Fig. 1b and c). According to these results, LG can reduce HD-induced NAFLD mice’s body weight gain rate, body fat ratio, and liver index. Compared to the NC group, the hepatic total cholesterol (TC) and total triglycerides (TG) levels increased significantly in HD, but the hepatic TC decreased significantly in LG (Fig. 1e). The hepatic TG and the serum free fatty acid (FFA) had a decreasing trend with LG administration (Fig. 1d and f). The hepatic HE staining sections of HD demonstrated severe micro/macrovesicular steatosis and frequent ballooning of hepatocytes, which were significantly ameliorated (Fig. 1g and h). HD liver tissue was stained with oil red O to reveal a significant reduction in liver lipid deposition after LG treatment (Fig. 1i and j). Compared to the NC group, the fasting blood glucose, fasting serum insulin, and homeostasis model assessment of insulin resistance (HOMA-IR) levels all increased significantly in HD, and the fasting blood glucose showed a downward trend in LG (Fig. 1k), while LG decreased the fasting serum insulin and HOMA-IR levels significantly (Fig. 1l and m). The levels of serum alanine aminotransferase (ALT), aspartate aminotransferase (AST), and hepatic malondialdehyde (MDA) in HD all increased significantly versus the NC group. Serum ALT and hepatic MDA were reduced by LG (Fig. 1n and p), and serum AST decreased in LG with near marginal significance (Fig. 1m). The above results showed that LG could ameliorate liver function and liver lipid deposition in mice fed HD.

### LG remodeled the gut microbiota structure of NAFLD mice

**Fig 2:**
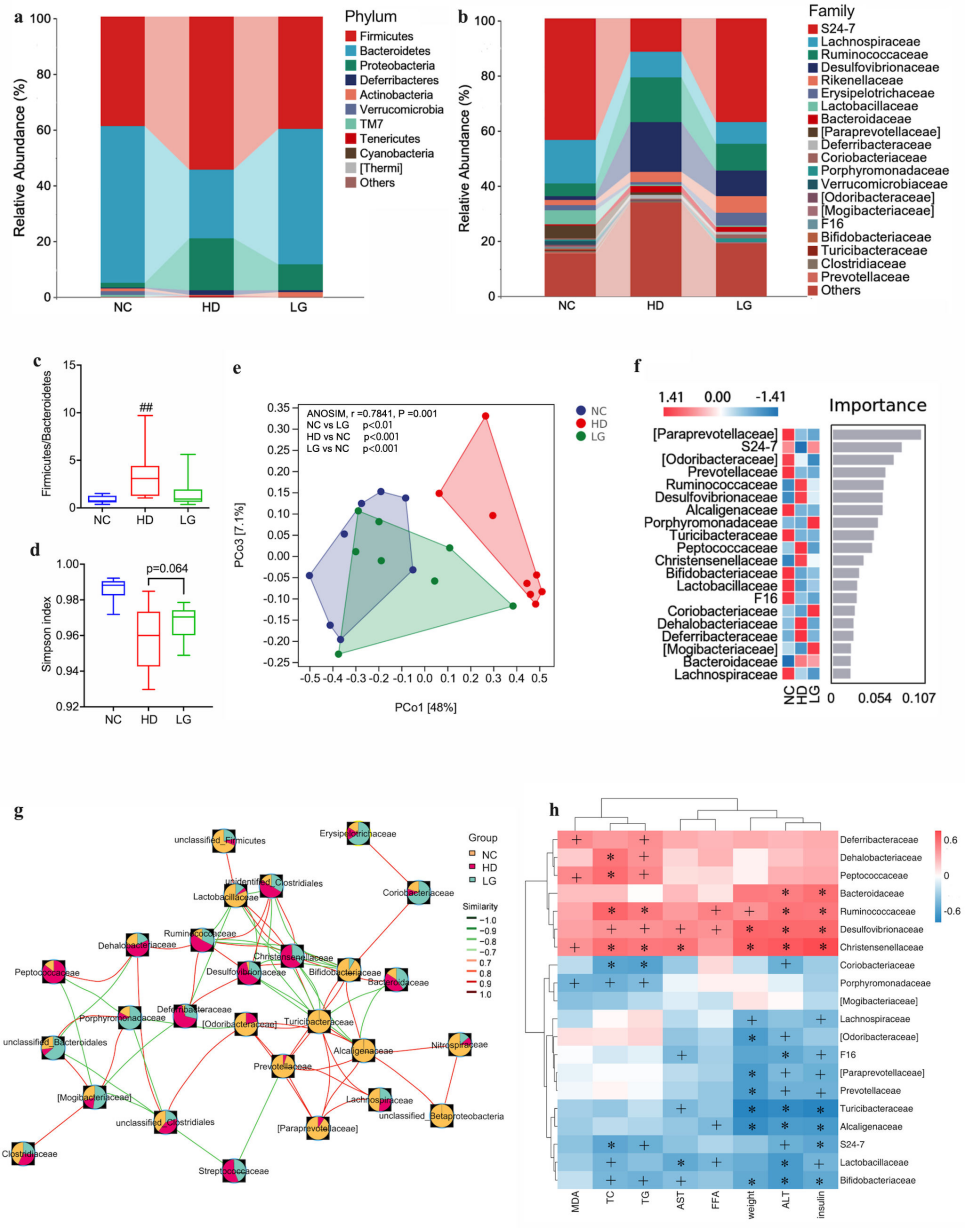
Effects of LG on the structure of gut microbiota. (a) Bacterial composition at the phylum level; (b) bacterial composition at the family level; (c) F/B ratio; (d) alpha-diversity analysis with the Simpson index; (e) principal coordinate analysis (PCoA) of three groups based on weighted UniFrac metrics, each spot representing one group with mean ± SD; (f) random forest analysis at the family level; (g) network analysis of highly abundant families with positive interactions in red and negative interactions in green. # *P* < 0.05, ## *P* < 0.01 compared with the NC group. *P < 0.05, **P < 0.01 compared with the HD group. Box plots display medians with interquartile ranges. (h) Spearman correlations between the gut microbial community at the family level and vital metabolic parameters related to NAFLD. + *P* < 0.05, *P < 0.01.

To examine whether the gut microbiota is involved in the improvement of NAFLD by LG, we sequenced the 16S rRNA gene. At the phylum level, the gut microbiota is dominated by *Firmicutes, Bacteroides*, and *Proteobacteria* (Fig. 2a). *Proteobacteria* are composed of numerous opportunistic pathogens, whose abundance increases significantly in the HD group and decreases significantly in the LG group. At the family level (Fig. 2b), LG showed a tendency to reverse HD-induced changes in bacterial composition, which were characterized by higher abundances of S24_7 and *Lachnospiraceae* with lower abundances of *Desulfovibrionaceae* and *Ruminococcaceae*. Moreover, the Firmicutes/Bacteroidetes (F/B) ratio increased significantly in HD but decreased in LG with a certain trend (*P* = 0.0833) (Fig. 2c).

Compared to the NC group, the Simpson index of HD reflected a trend toward less diversity, while there was a recovery tendency in the diversity of LG close to statistical significance (*P* = 0.064) (Fig. 2d). The gut microbiota structure was significantly different among three groups (analysis of one-way similarity (ANOSIM), r = 0.7841, *P* = 0.001). Furthermore, β-diversity based on weighted UniFrac distances showed differences in microbiome composition between LG and HD, while LG had a similar diversity with NC (Fig. 2e). To identify key bacteria, we conducted a differential analysis using random forest analysis and the Wilcoxon rank-sum test. In total, 20 families were identified as discriminating families in the three groups via random forest analysis, and *S24-7*, *Ruminococcaceae*, and *Desulfovibrionaceae* were ranked as discriminative families (Fig. 2f). After analyzing the network of high-abundance families, we investigated how the microbial communities interacted. The NC group showed a cluster of strongly related bacteria *Bifidobacteraceae*, *Lactobacilaceae*, *Alcaligenaceae*, and *Turicibacteraceae*, while LG could recuperate the portion of them under the adverse impact of HD. The enriched bacteria, such as *Desulfovibrionaceae*, *Ruminococcaceae,* and *Dehalobacteriaceae* were associated, whereas LG reduced the enrichment of these bacteria (Fig. 2g). Spearman’s correlation heatmap was used to indicate the relations between major bacteria and significant metabolic biomarkers related to NAFLD (Fig. 2h). These bacteria enriched in HD, such as *Ruminococcaceae*, *Desulfovibrionaceae*, and *Christensenellaceae*, were positively correlated with metabolic biomarkers related to NAFLD. However, these bacteria enriched in NC and LG, such as *S24_7*, *Bifidobacteriaceae*, and *Lactobacillaceae*, were negatively correlated with metabolic biomarkers related to NAFLD, including body weight, insulin, hepatic TC, TG, and FFA, and serum levels of ALT and AST.

### LG modulated the bacterial metabolism of NAFLD mice

**Fig 3:**
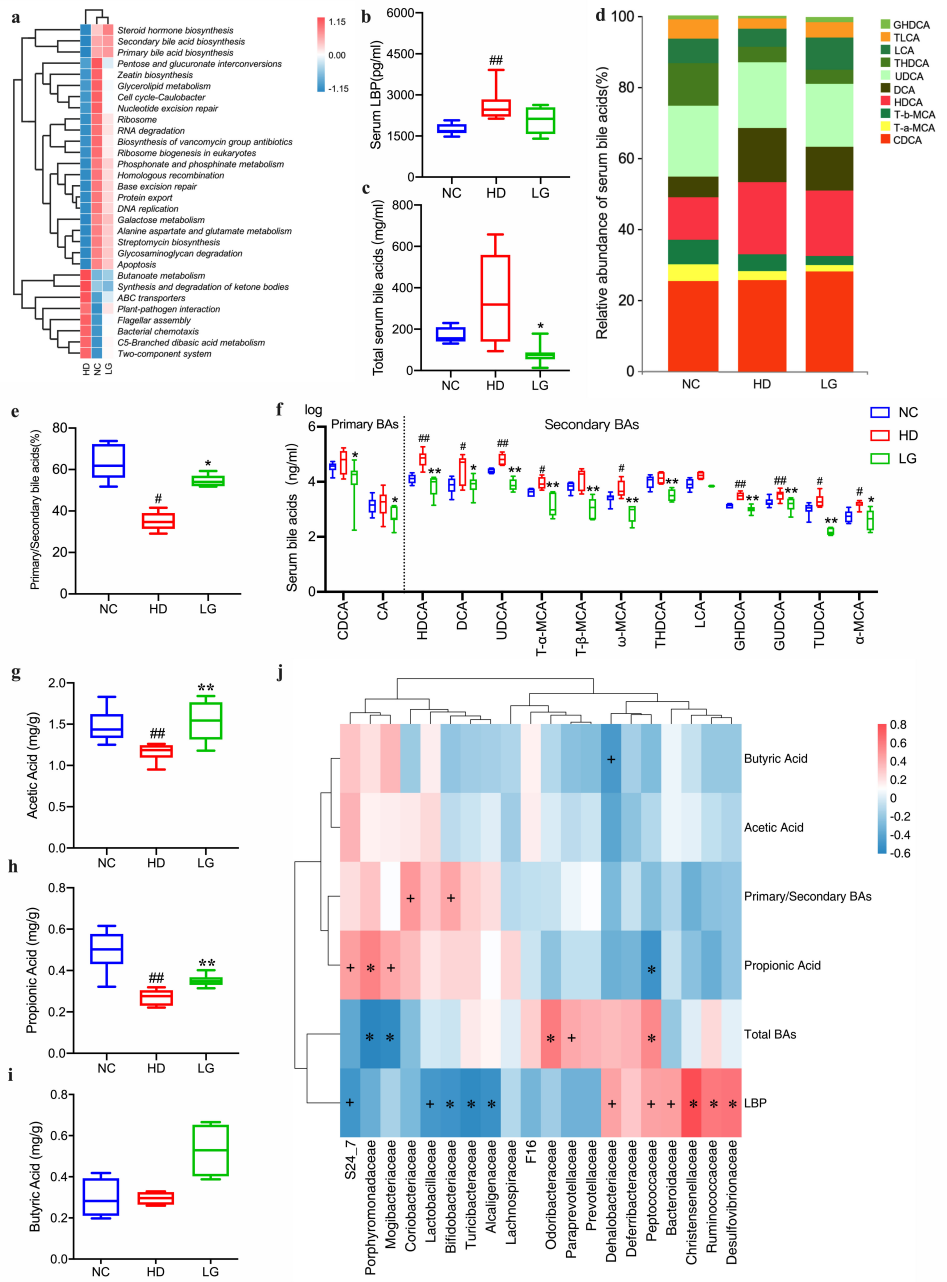
Effects of LG on the metabolites of gut microbiota. (a) Heatmap of different pathways between groups; (b) concentration of lipopolysaccharide-binding protein (LBP) in serum; (c) concentration of total BAs in serum; (d) relative abundance of different BAs in serum; (e) the ratio of primary/secondary BAs in serum; (f) high and low abundantly different components of BAs in serum; concentrations of acetic acid (g), propionic acid (h), and butyric acid (i) in feces. # *P* < 0.05, ## *P* < 0.01 compared with the NC group. *P < 0.05, **P < 0.01 compared with the HD group. Box plots display medians with interquartile ranges. (j) Spearman correlations between the gut microbial community at the family level and their major metabolites. + *P* < 0.05, *P < 0.01.

Phylogenetic Investigation of Communities by Reconstruction of Unobserved States (PICRUSt) analysis was performed to predict the gut microbiota’s function. Through statistical algorithms, 30 kyoto encyclopedia of genes and genomes (KEGG) pathways showed significant differences (Fig. 3a). Analysis of the clustering of the 30 KEGG pathways indicated that the LG group clustered with the NC group, which was analogous to the results of PCoA (Fig. 2e). We found that the pathways of steroid hormone biosynthesis and primary and secondary BAs biosynthesis were higher in the NC and LG groups, whereas the pathways of butanoate metabolism, synthesis, and degradation of ketone bodies were lower in NC and LG. These results suggested that LG could restore the functional profile of the HD-induced gut microbiota to the NC.

To confirm the results of PICRUSt, we examined bacteria-associated metabolites. Compared to the NC group, the concentration of lipopolysaccharide-binding protein (LBP) in serum was increased significantly in HD and decreased in LG at the margin of statistical significance (*P* = 0.0692) (Fig. 3b). The concentration of total BAs in serum increased significantly in HD and decreased significantly in LG (Fig. 3c).

Subsequently, we analyzed the specific components of the BAs in serum and found that the relative abundances of deoxycholic acid (DCA) and hyodeoxycholic acid (HDCA) were increased in HD compared to NC and were decreased in LG (Fig. 3d). Nevertheless, the ratio of primary/secondary BAs in serum decreased significantly in HD and increased significantly in LG (Fig. 3e).

We compared the levels of different BA components in serum between groups. Compared to the NC group, HDCA, DCA, ursodeoxycholic acid (UDCA), tauro-α-muricholic acid (T-α-MCA), ω-muricholic acid (ω-MCA), glycohyodeoxycholic acid (GHDCA), glycoursodeoxycholic acid (GUDCA), tauro ursodesoxy cholic acid (TUDCA), and α-muricholic acid (α-MCA) showed a significant increase in HD. The chenodeoxycholic acid (CDCA), cholic acid (CA), HDCA, DCA, UDCA, T-α-MCA, tauro-β-muricholic acid (T-β-MCA), ω-MCA, taurohyodeoxycholic acid, GUDCA, α-MCA, and ω-MCA in LG showed a significant decrease compared to HD (Fig. 3f). Based on the above, we found that total serum BAs can be increased by HD, but the majority of secondary BAs components, which are derivatives of primary BAs, were reduced after LG treatment.

Compared to the HD group, the concentrations of acetic acid, propionic acid, and butyric acid in feces all increased significantly in LG (Fig. 3g through i). Compared to the NC group, the concentrations of acetic acid and propionic acid in feces decreased significantly in HD, while the concentration of butyric decreased slightly in HD (Fig. 3i). Spearman’s correlation heatmap showed the relations between major bacteria and their significant metabolites related to NAFLD (Fig. 3j). These bacteria enriched in the HD, such as *Peptococcaceae*, *Dehalobacteriaceae*, *Odoribacteraceae*, *Ruminococcus*, and *Paraprevotellaceae* had a positive relationship with total BAs but a negative relationship with SCFAs, such as acetic acid, propionic acid, and butyric acid. However, bacteria enriched in NC and LG, such as *S24_7* and *Mogibacteriaceae*, had a positive relationship with propionic acid and primary/secondary BAs but a negative relationship with BAs.

### LG-mediated therapeutic effects via molecular pathways

**Fig 4:**
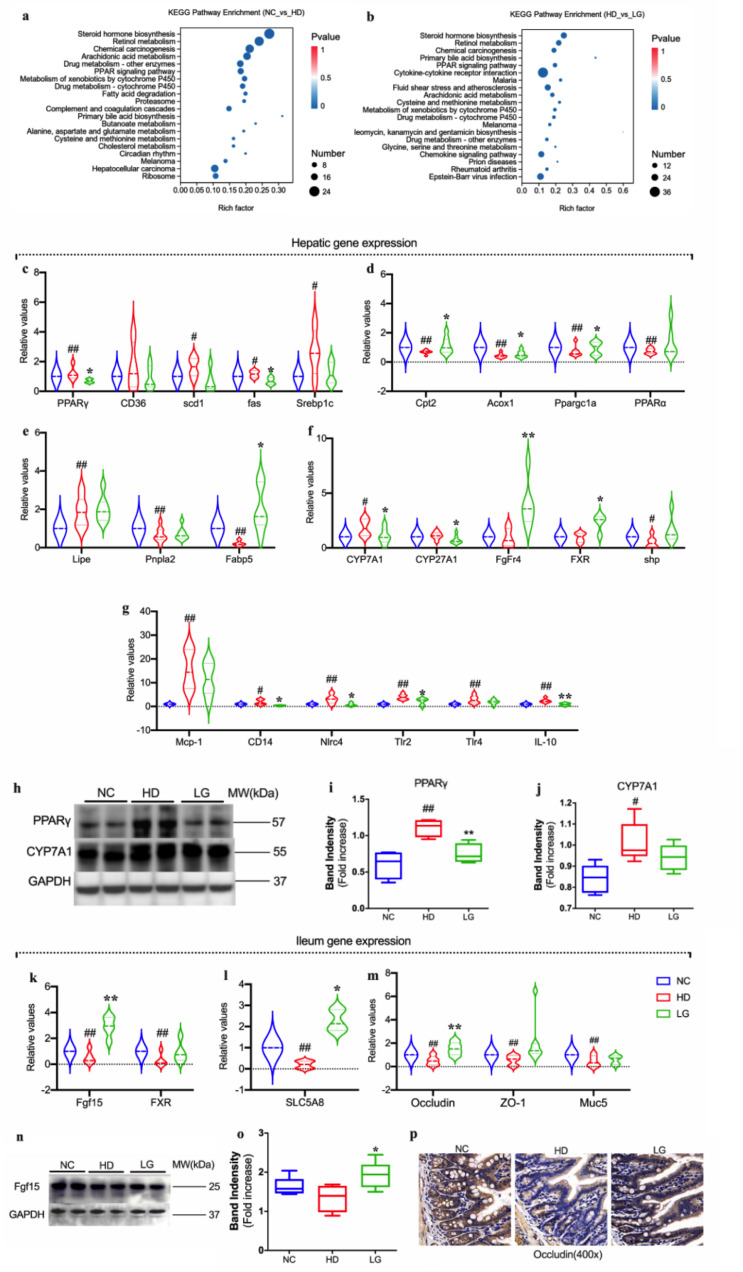
Potential molecular mechanism of LG in the treatment of NAFLD. (a) Enrichment analysis of KEGG metabolic pathway for the NC group in comparison with that of the HD group; (b) enrichment analysis of KEGG metabolic pathway for the LG group in comparison with that of the HD group; compared with the NC group, the relative expression of genes encoding for lipogenesis (c), fatty acid oxidation (d), lipolysis (e), inflammation (f), BAs biosynthesis (g) in the liver; (h) Western blotting analysis of PPARγ, CYP7A1 protein expression in the liver; cumulative densitometric analysis for PPARγ (i),CYP7A1 (j)in the liver of representative mice per each group. Band densities were normalized to the expression level of glycerol triphosphate dehydrogenase (GAPDH). Compared with the NC group, the relative expression of genes encoding for BAs biosynthesis (k), SCFAs receptor (l), intestinal permeability (m) in ileum; (n) Western blotting analysis of Fgf15 protein expression in the ileum; (o) cumulative densitometric analysis for Fgf15 in the ileum of representative mice per each group. Band densities were normalized to the expression level of GAPDH. (p) Immunohistochemical staining for Occludin protein expression in the terminal ileum (magnification, ×400). # *P* < 0.05, ## *P* < 0.01 compared with the NC group. *P < 0.05, **P < 0.01 compared with the HD group. Violin and Box plots display medians with interquartile ranges.

Based on the annotated genomes and genes differentially expressed between NC versus HD and HD versus LG, KEGG functional enrichment analysis was conducted (Fig. 4a and b).

Compared to the NC group, HD is related to the peroxisome proliferator-activated receptor (PPAR) signaling pathway, primary BA biosynthesis, and the butanoate metabolism signaling pathway. Compared to the HD group, LG could regulate the PPAR signaling pathway, cytokine‒cytokine receptor interaction, and primary BA biosynthesis signaling pathways. Reverse transcription PCR (RT-PCR) analysis was performed to verify the transcriptome results on the metabolism of lipids, BAs, SCFAs, and inflammatory signaling pathways. The expression of genes related to hepatic lipid synthesis (PPARγ, CD36, scd1, fas, Srebp1c), genes related to lipolysis (Lipe, Pnpla2, Fabp5), genes related to lipid oxidation (Cpt2, Acox1, Ppargc1a, PPARα), genes related to BAs biosynthesis (CYP7A1, CYP27A1, FgFr4, FXR, shp), and genes related to hepatic inflammation (Mcp-1, CD14, Nlrc4, Tlr2, Tlr4, IL-10) in liver tissues was measured by RT-PCR. The expression of genes related to BAs biosynthesis (Fgf15, FXR), a gene related to SCFAs (SLC5A8), and genes related to intestinal permeability (Occludin, ZO-1, Muc5) in ileal tissue was also measured by RT-PCR (Fig. 4c through g and i through k). Compared to the NC group, the expression of hepatic lipogenesis genes PPARγ, hepatic bile acid biosynthesis genes CYP7A1, hepatic inflammation genes Mcp-1, Tlr2, Tlr4, IL-10 upregulated significantly, while the expression of hepatic lipid oxidation genes Cpt2, Acox1, Ppargc1a, hepatic lipolysis gene Lipe, Pnpla2 and Fabp5, ileum SCFAs receptors SLC5A8, and intestinal permeability gene Occludin and ZO-1 downregulated significantly in HD.

However, compared to the HD group, the expression of hepatic BAs biosynthesis genes CYP27A1, hepatic inflammation genes CD14, Nlrc4, and IL-10 was downregulated significantly, while the expression of hepatic lipolysis gene Fabp5, ileum BAs biosynthesis gene Fgf15, intestinal permeability gene ZO-1, and ileum SCFAs receptors SLC5A8 was upregulated significantly in LG. The higher gene expression of PPARγ and CYP7A1 in the livers of HD and the higher gene expression of Fgf15 in the ileum of LG were verified by Western blot (Fig. 4h through j, n and o). The higher gene expression of Occludin in the ileum of LG was verified by immunohistochemistry (Fig. 4p).

## DISCUSSION

**Fig 5:**
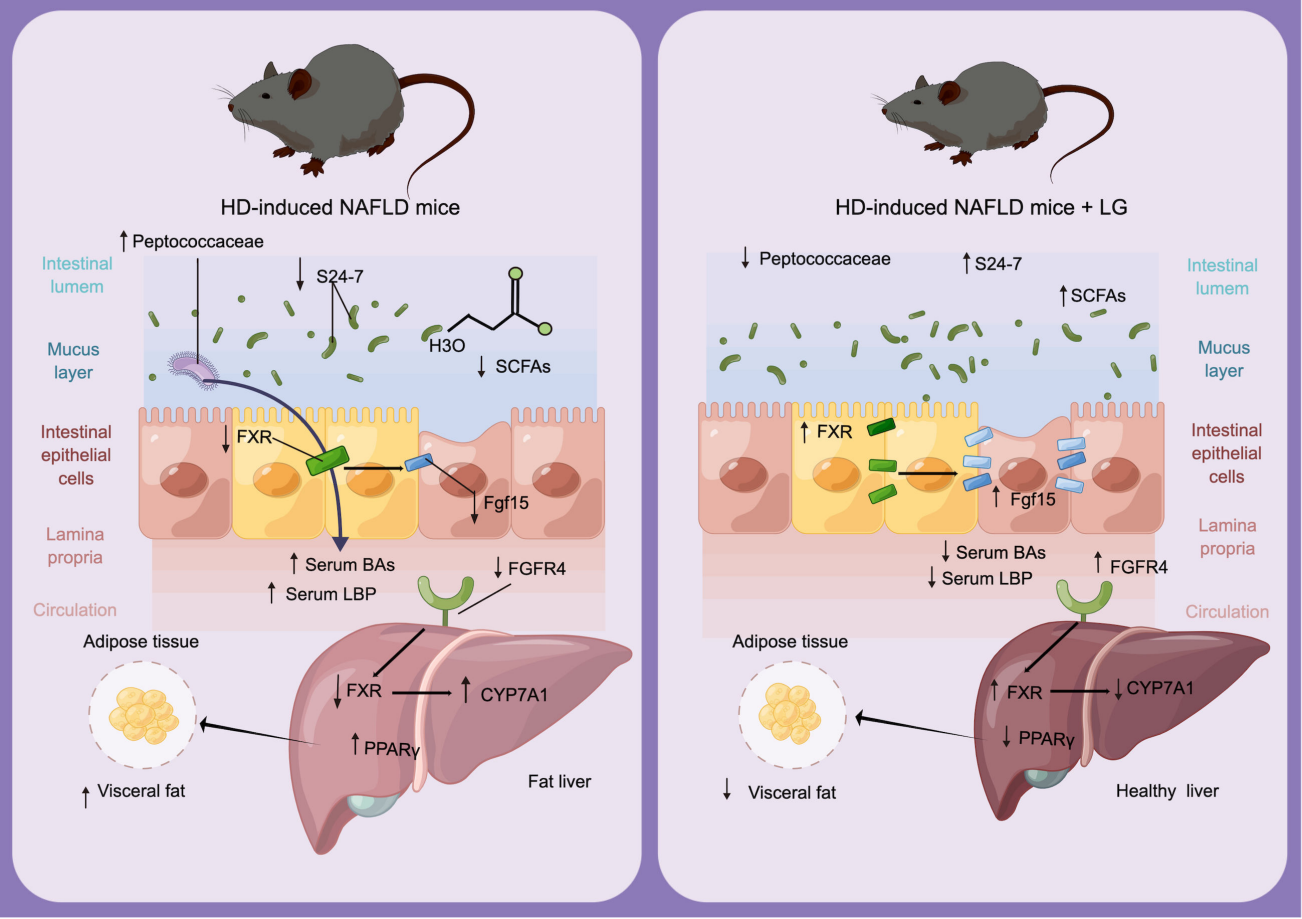
The proposed molecular mechanism by which LG ameliorates NAFLD. Left: The administration of HD increased the abundance of *Peptococcaceae* and decreased the expression of FXR in both the ileum and liver. Consequently, the activation of CYP7A1 in the liver was observed, leading to an accelerated biosynthesis of BAs. Additionally, HD caused a reduction in the abundance of S24-7 and inhibited the production of SCFAs. Furthermore, HD induced an increase in the expression of PPARγ in the liver, thereby promoting lipid synthesis. Right: The administration of LG resulted in a reduction in the abundance of *Peptococcaceae* and activation of FXR expression in both the ileum and liver. Consequently, the expression of CYP7A1 in the liver was suppressed, leading to the inhibition of BAs biosynthesis. Additionally, LG supplementation increased the abundance of S24-7 and facilitated the production of SCFAs. Furthermore, LG administration decreased the expression of PPARγ in the liver, thereby reducing lipid synthesis.

The potential of TCM as a treatment for NAFLD has been proposed, however, the lack of understanding regarding its mechanism poses a hindrance to the advancement and promotion of TCM. In this research, it was discovered that LG treatment effectively decreased body weight and fat accumulation in NAFLD mice induced by HD, while also improving liver steatosis, inflammation, and intestinal barrier function through the modulation of disrupted gut microbiota. Additionally, LG was found to ameliorate NAFLD by elevating the concentration of SCFAs in the intestine and reducing the concentration of BAs in the serum. The summarized outcome of LG’s effectiveness in ameliorating NAFLD is depicted in Fig. 5.

Gut microbiota has recently emerged as a significant target for TCM to treat NAFLD. In a recent animal study, it was found that LG could inhibit the abundance of *Peptococcaceae* and *odoribacter* in rats with nonalcoholic steatoheoatitis (NASH) (17), which aligns with our funding. Previous research has also indicated that LG can increase the abundance of *Lactobacillus* in mice and rats with HD diets (18, 19), which is consistent with our results. Additionally, another animal study reported that LG could increase the abundance of *Alistipes putredinis*, a known butyrate producer, in mice with HD-induced hepatosteatosis (20). Liquiritin, a prominent constituent of LG, has been demonstrated to possess antioxidant properties. Additionally, the presence of beneficial gut microbiota has been found to enhance the bioactivity of liquiritin (21). Previous studies have provided initial evidence regarding the alterations in gut microbiota following LG intervention. However, there is a dearth of research investigating the functional capacity of gut microbiota in response to LG treatment. Through the utilization of PICRUSt analysis, our study has identified that LG has the potential to modulate the biosynthesis pathway of BAs and the metabolism pathway of butanoate. These findings have been further validated at both the metabolite and molecular levels.

BAs serve as crucial signaling molecules, exerting significant influence on the body’s energy metabolism and inflammatory response, thereby establishing a strong association with liver diseases (22). By prior findings in a study involving patients with NASH (15), our investigation revealed a notable reduction in the ratio of primary to secondary BAs in mice with NAFLD. However, following treatment with LG intervention, there was a discernible increase in this aforementioned ratio (23). The findings of our study indicate that LG treatment effectively mitigated the bile acid metabolism disorder associated with NAFLD, as evidenced by the significant reduction observed in the levels of various BAs, including CDCA, HDCA, DCA, UDCA, T-α-MCA, T-β-MCA, ω-MCA, GHDCA, GUDCA, TUDCA, and α-MCA. CDCA is a major primary BA that has been proven to promote liver fibrosis (24). Secondary BAs are higher in obese NAFLD patients with extensive fibrosis, specifically LCA and DCA (25). While one study suggests that UDCA may yield positive effects on NAFLD (26), another study indicates that oral UDCA treatment leads to an augmentation in the synthesis of hepatotoxic abnormal BAs (27). The gut microbiota may undergo alterations due to BAs, while the activity of the gut microbiota can also impact the bile acid pool (28). Our correlation results further showed that *Peptococcaceae* is positively related to total BAs. *Peptococcaceae* has been reported to be involved in the oxidation and isomerization of the bile acid hydroxyl Groups C3, C7, and C12 (29), positively associated with obesity, type 2 diabetes, and NAFLD (30). The inhibition of ileum Fgf15 and FXR expression, as well as the activation of hepatic CYP7A1 expression, were observed following HD treatment. Conversely, the activation of FXR expression was observed, subsequently leading to the inhibition of CYP7A1 expression in the liver after LG treatment. The activation of CYP7A1 promotes hepatic bile acid biosynthesis and biliary cholesterol secretion and contributes to the development of NAFLD (31, 32). Our study suggested that LG could influence bile acid metabolism by adjusting the gut microbiota to attenuate NAFLD.

SCFAs, mainly consisting of acetic acid, propionic acid, and butyric acid, are the major metabolites produced in the colon by bacterial fermentation and maintain homeostasis of the gut environment (33). Previous studies have shown that *Astragalus polysaccharides* exhibit anti-NAFLD effects by enriching *Desulfovibrio vulgaris* in the gut microbiota to produce acetic acid (34). The increased concentrations of acetic acid, propionic acid, and butyric acid after LG treatment suggested that LG could promote SCFA production. Acetic acid could lower abdominal fat accumulation and protect against the accumulation of lipids in the liver (35), propionic acid could protect against HD-induced obesity, inflammation, and insulin resistance and promote satiety in obese subjects (36), and butyric acid is known to inhibit inflammation (37). Our result also showed that *Porphyromonadaceae*, *Mogibacteriaceae*, and *S24_7* have positive relationships with SCFAs. It has been found that *Porphyromonadaceae* could modulate adiposity by producing SCFAs to reduce visceral fat mass (38). Notably, we found that LG could increase the expression of SLC5A8, a monocarboxylate transporter in the gastrointestinal tract that facilitates the entrance of SCFAs into normal cells (39). The findings of our study indicate that LG has the potential to modulate the composition of gut microbiota, leading to increased production of SCFAs and subsequently mitigating the development of NAFLD induced by HD.

Moreover, our research initially investigated the potential mechanism through which LG mitigates hepatic lipid synthesis in the treatment of NAFLD. PPARγ is a master regulator of whole-body lipid metabolism, adipogenesis, and insulin sensitivity (40), while PPARα controls genes involved in fatty acid uptake, lipid deposition, and β-oxidation (41). Therefore, we examined PPARγ, PPARα, and their target genes, CD36, fas, Srebp1c, and Fabp5 in the liver. The findings of our study indicate that HD treatment resulted in an upregulation of PPARγ, CD36, and fas expression while downregulating the expression of PPARα and Fabp5 in the liver. Conversely, LG treatment exhibited a reversal of this trend. These results suggest that LG has the potential to mitigate lipid accumulation in NAFLD by reducing lipogenesis and enhancing lipid oxidation. These results suggested that LG could reduce the accumulation of lipids in NAFLD to a certain extent by reducing lipogenesis and increasing lipid oxidation.

LG may potentially improve NAFLD by suppressing the growth of bile acid-producing bacteria and promoting the growth of short-chain fatty acid-producing bacteria. Additionally, LG has been found to decrease lipogenesis in NAFLD mice by modulating the PPAR signaling pathway, thereby reducing hepatic inflammation and enhancing intestinal barrier function. The findings of our study provide valuable insights into the treatment of NAFLD and offer the potential for the development of novel therapeutic prebiotics utilizing Chinese herbal compounds.

## MATERIALS AND METHODS

### The preparation of LG decoction

The components of LG are listed in Table 1. Sichuan Neo-Green Pharmaceutical Technology Development Co. Ltd. supplied all herbs. LG was administrated at the standard dose (715 mg/kg/d) in mice, which is equivalent to that given to human patients in clinical studies (9, 42). HPLC was applied to analyze the ingredients of LG according to methods developed by the Hong Kong Chinese Materia Medica Standards (43) (Fig. S1).

**TABLE 1:** The composition of LG

Chinese name	Scientific name	Botanical name	Medicinal part	Weight (g)
Fulin	Poria	*Wolfiporia cocos* (*F.A. Wolf*) *Ryvarden & Gilb.*	Sclerotium	12
Guizhi	Ramulus Cinnamom	*Cinnamomum cassia Presl*	Twig	9
Baizhu	Rhizoma Atractylodis Macrocephalae	*Atractylodes macrocephala Koidz.*	Rhizome	6
Gancao	Radix Glycyrrhiza	*Glycyrrhiza uralensis Fisch.*	Root & rhizome	6

### Animal study

The Shanghai Research Center of Southern Model Organisms (Shanghai, China) provided 6- to 8-week-old male C57BL/6 mice that were group-housed (3–4 per cage) in specific pathogen-free conditions. The Animal Ethics Committee at Shanghai University of Traditional Chinese Medicine authorized all animal experiments in this work (approval number PZSHUTCM210512413, 12 December 2019), which were carried out according to the “Guide for the Care and Use of Laboratory Animals” approved by the US National Institutes of Health.

We randomly divided mice into two groups after 1 week of acclimatization: one group was given a normal chow diet (NC, *n* = 10) and the other group was given a high-fat diet (HD, *n* = 18). Two mice per group were dissected randomly after 16 weeks on an HD diet to determine the success of the NAFLD mouse model. The HD group was then separated into two groups, each with eight mice: one group received HD and 0.5% sodium carboxymethyl cellulose intragastrically once daily; the other group received HD and LG intragastrically once daily. All animals were euthanized under anesthesia with Pentobarbita (100 mg/kg, intraperitoneal injection) after 8 weeks of treatment, and blood, liver, and intestinal tissues were collected according to strict protocols.

### Serum and hepatic biochemical analyses

The Catalyst One Chemistry Analyser (IDEXX Laboratories) was used to measure the levels of serum ALT and AST. Commercial kits were used to measure total TG, TC, FFAs, and MDA in extracted hepatic samples (Nanjing Jiancheng Bioengineering Institute, A110-1-1, A111-1-1, A042-2-1, A003-1-2, respectively). The serum insulin and LBP levels were measured with ELISA kits (Crystalchem, 90080; Abcam, ab269542, respectively).

### Histopathology of the liver

Mouse liver tissues were extracted and preserved for 48 hours in a 10% formalin solution. Haematoxylin and eosin staining was performed after the tissues were dehydrated, cleaned, waxed, embedded, sliced, and embedded in plastic. Oil red O staining of frozen liver slices confirmed the diagnosis of steatosis. Under a light microscope, the morphological differences of the tissues were observed, and all quantification was completed by Image-Pro Plus software 6.0.

### Analysis of gut microbiota

Using Illumina high-throughput sequencing, we evaluated the composition and functional profile of the gut microbiota by sequencing the 16S rRNA gene V3–V4 region in mouse feces. Using the DNeasy PowerSoil Kit, total bacterial DNA was isolated from mouse feces (QIAGEN, Inc., the Netherlands). The forward primer 338F (5′-ACTCCTACGGGAGGCAGCA-3′) and the reverse primer 806R (5′GGACTACHVGGGTWTCTAAT-3′) were used to generate an amplicon library for double-ended (2 × 300 bp) sequencing with the Illumina MiSeq platform. The PicoGreen dsDNA Assay Kit was used to quantify PCR amplicons purified using Agencourt AMPure Beads (Beckman Coulter, Indianapolis, IN; Invitrogen, Carlsbad, CA, USA). Amplicons were pooled in equal numbers once they had been quantified. The raw readings were combined after they were quality-filtered. FLASH was used to construct paired-end readings. After chimera detection, the remaining sequences were used. UCLUST grouped the remaining high-quality sequences into operational taxonomic units (OTUs) at 97% sequence identity after chimera identification. Using default settings, a typical sequence was chosen from each OTU. The representative sequences were BLAST searched against the Greengenes database, and the best hit was used for OTU taxonomic categorization. An averaged, rounded rarefied OTU table was constructed by averaging 100 uniformly resampled OTU subsets under 90% of the minimum sequencing depth for subsequent analysis to reduce the variation in sequencing depth among samples. Overall samples, OTUs with less than 0.001% of the sequences were filtered. PICRUSt2 software (https://github.com/gavinmdouglas/q2-picrust2/releases/tag/2021.11_0) (44) was used to predict the functional potential of the microbial community. Metabolic pathways were annotated using the KEGG database (https://www.kegg.jp/) (45).

### Fecal SCFAs quantification

Gas chromatography/mass spectrometry (GC/MS) was used to determine the concentration of SCFAs in the feces from the caecum as previously reported (46). In summary, 50 mg of fecal material was homogenized in 0.8 mL ddH_2_O, followed by 0.01% H_2_SO_4_ and 1 mL diethyl ether. After vortexing for 30 seconds, the mixture was centrifuged for another 5 minutes at 4°C and 10,000 rpm. Two hundred microliters of supernatant were collected using an Agilent 7000B system (Agilent Technologies, CA, USA), equipped with flame ionization and thermal conductivity detectors, capillary columns, and GC ChemStation software. Pure standards diluted in diethyl ether were used to measure acetate, butyrate, propionate, valerate, isobutyrate, and isovalerate.

### Serum BAs measurement

Liquid chromatography/mass spectrometry (LC/MS) was used to determine the concentration of BAs in the serum as previously reported (47). The separation of BAs was conducted using an Acquity UPLC BEH C18 column (2.1 × 100 mm, 1.7 µm) (Waters, Milford, MA, USA). The sample injection volume was 5 µL, with a column temperature of 45°C. The mobile phase consisted of formic acid (0.01% in water, solvent A) and acetonitrile (solvent B), at a flow rate of 0.3 mL/minute. The gradient elution program was as follows: 0–1 minute, 55% B; 1–9 minutes, 55%–80% B; 9–11.4 minutes, 80%–90% B; 11.4–14 minutes, 90%–90% B; 14–14.1 minutes, 90%–55% B. The samples were analyzed by a mass spectrometer in negative electrospray ionization under a multiple-reaction mode. Operating parameters were ion source temperature: 120°C; desolventizing temperature: 350°C; desolventizing gas (N2) flow rate: 600.0 L/hour; cone gas (N2) flow rate: 5.0 L/hour; capillary voltage: 2.8 kV; cone voltage: 55 V. Data for the determination of BAs were analyzed and processed using Analyst Software 1.6 (Agilent).

### RNA sequencing

Total RNA was selected from three randomly chosen NC, HD, and LG livers (*n* = 3). The RNA Seq library was constructed using the Illumina TruSeq RNA Sample Preparation Kit according to the manufacturer’s instructions. The library was then enriched for segments 300–400 bp in length using PCR amplification. The library quality was then verified using an Agilent 2100 Bioanalyzer. These libraries were then sequenced at paired ends using Next-Generation Sequencing on the Illumina HiSeq sequencing platform (Personalbio, Shanghai, China). We then utilized HTSeq to compare each gene’s read count value to the original expression of that gene using fragments per kilobase million (FPKM) to optimize the expression.

### Reverse transcription PCR

Total RNA was extracted from liver and colon tissue and reverse transcribed into cDNA utilizing the SuperScriptTM First-Strand Synthesis System (Invitrogen). A LightCycler96 (Hangzhou Bioer Technology, Share-Holding Co.), and iQ SYBR Green Supermix (BIO-RAD, USA) were used for RT-PCR. Genes related to hepatic lipid synthesis (Scd1, fas, PPARγ, CD36), genes related to lipolysis (Lipe, Pnpla2, Fabp5), genes related to lipid oxidation (Cpt2, Acox1, Ppargc1a, PPARα), genes related to bile acid transporters (shp, CYP7A1, CYP27A1, FgFr4), genes related to hepatic BAs biosynthesis (FXR), genes related to ileal bile acid transporters (Fgf15), genes related to liver inflammation (CD14, TLR2, TLR4, NLRC4, MCP-1, IL-10), genes related to intestinal permeability (Occludin, ZO-1, Muc5), and genes related to short-chain fatty acids (SLC5A8) were all adjusted with glycerol triphosphate dehydrogenase (GAPDH) as a housekeeping gene. Relative quantification was calculated using the −∆∆Ct method. The primer sequences for RT-PCR can be found in Table S1.

### Western blot analysis of hepatic and ileal proteins

To extract total protein, mouse liver tissues were lysed in RIPA buffer with one protease and phosphatase inhibitor cocktail. The BCA technique was used to determine the total protein content in the supernatant. After denaturation for 5 minutes at 100°C, equivalent quantities of proteins were run on a 10% SDS-PAGE gel and transferred to a polyvinylidene fluoride (PVDF) membrane. The membrane was blocked for 2 hours at room temperature with 5% nonfat milk in 0.1% tris-buffered saline with tween-20 (TBST) before being incubated with a primary antibody overnight at 4°C. After thorough washing, the membranes were incubated for 2 hours at room temperature with the appropriate HRP-conjugated secondary antibody. Finally, an ECL reagent was used to develop the blots. The primary antibodies listed below were used: GAPDH [monoclonal antibody (2B8), YM3029, 1:5,000], PPARγ (polyclonal antibody, YM33072, 1:1,000), CYP7A1 (polyclonal antibody, NBP-04836, 1:500), and Fgf15 (polyclonal antibody, ab229630, 1:500). The quantitative image analysis was performed using Image J (2.0.0 version, National Institutes of Health, Bethesda, MD, USA).

### Immunohistochemistry

Intestinal Occludin expression was evaluated using paraffin-embedded liver and ileum tissues. After deparaffinization, the slides were heated in an autoclave with sodium citrate for antigen repair, followed by 1% hydrogen peroxide to abolish 10 endogenous peroxidase activity, and blocked with 2% goat serum. Slides were then incubated with primary antibodies, including Occludin (Servicebio, GB111401, 1:500), at 4°C overnight. HRP-conjugated secondary antibodies (1:200) were incubated for 50 minutes at room temperature. After washing with PBS, the peroxidase substrate DAB (Dako, K5007) was used for color development.

### Statistical analysis

The results are displayed as the mean ± standard deviation. The graphs depict the data distribution in the form of box and violin graphs. The Mann‒Whitney U test was used to examine significant differences in biochemical markers, F/B, and α-diversity of gut microbiota, across groups in GraphPad Prism 8.4 software. Significant differences in the microbiota structure among the three groups were evaluated by nonparametric analysis of similarity (ANOSIM, permutations = 9,999) using the vegan package in R (version 3.6.1). Permutational multivariate analysis of variance permutational multivariate analysis of variance (PERMANOVA; permutations = 9,999) was used to assess the structural difference between the two groups (vegan package in R). Statistical significance was defined as *P* < 0.05. Differential abundance of pathways was identified by using the Kruskal–Wallis test. Benjamini–Hochberg method was used to adjust *P* values generated by multiple comparisons from PERMANOVA.

## Data Availability

The original sequences obtained in this investigation have been deposited in the NCBI sequence read archive (SRA) with the accession number PRJNA757473.

## SUPPLEMENTAL MATERIAL

Figure S1 spectrum.01979-23-s0001.pdf HPLC-based chemoprofile of LG.

Supplemental file 1 spectrum.01979-23-s0002.xlsx Results of multiple comparisons correction about biochemical indicators.

Supplemental file 2 spectrum.01979-23-s0003.xlsx Results of multiple comparisons correction about the concentrations of BAs and SCFAs between groups.

Supplemental file 3 spectrum.01979-23-s0004.xlsx Results of multiple comparisons correction about hepatic gene expression.

Supplemental file 4 spectrum.01979-23-s0005.xlsx Results of multiple comparisons correction about ileum gene expression.

Table S1 spectrum.01979-23-s0006.pdf Primer sequences for RT-PCR.

ASM does not own the copyrights to Supplemental Material that may be linked to, or accessed through, an article. The authors have granted ASM a non-exclusive, world-wide license to publish the Supplemental Material files. Please contact the corresponding author directly for reuse.
