# Supplementary material for: Ling-Gui-Zhu-Gan decoction ameliorates nonalcoholic fatty liver disease via modulating the gut microbiota
Source: Microbiol Spectr. 2024 Apr 22;12(6):e01979-23. doi: 10.1128/spectrum.01979-23 (PMC11237417; doi:10.1128/spectrum.01979-23)
Supplement: Figure S1 — HPLC-based chemoprofile of LG. [file spectrum.01979-23-s0001.pdf]

HPLC-based chemoprofile of Ling-Gui-Zhu-Gan Decoction

The sample was separated using an Agilent HPLC C18 column (4.6 × 250 mm, 5μm) maintained at a temperature of 30℃. The separation was achieved by employing a mixed mobile phase consisting of acetonitrile (A) and 0.05% formic acid in pure water (B). A gradient elution program was utilized, with the following time intervals and corresponding percentages of acetonitrile: 0 – 6 min, 3% – 19% A; 6 – 14 min, 19% A; 14 – 22 min, 19% – 22% A; 22 – 29 min, 22% – 30% A; 29 – 32 min, 30% A; 32 – 53 min, 30% – 51% A; 53 – 70 min, 51% – 90% A; 70 – 75 min, 90% A. The flow rate, detection wavelength, and injection volume were set at 1.0 mL/min, 265 nm, and 10 μL, respectively.

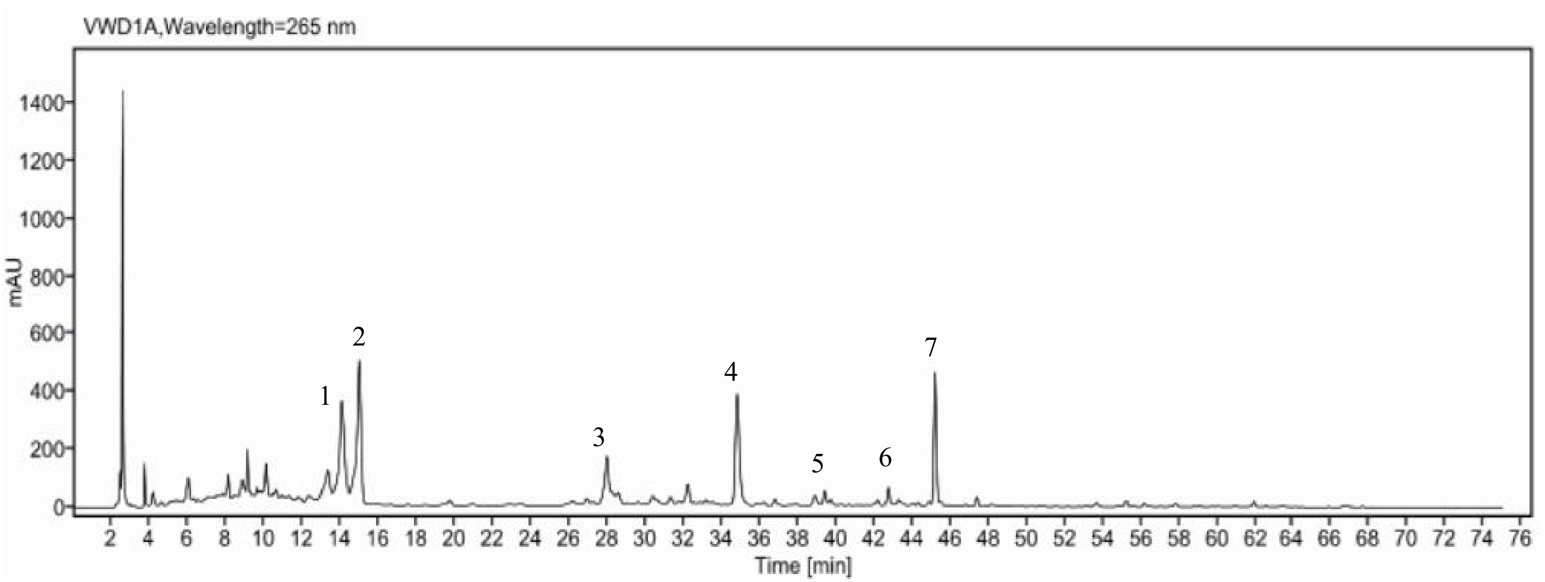

Figure S1: 1.liquiritin apioside; 2.liquiritin; 3.isoliquiritin; 4.cinnamic acid; 5.cinnamal dehyde; 6.licoricesaponin G2; 7.ammonium glycyrrhizinate
