# Supplementary material for: Ling-Gui-Zhu-Gan decoction ameliorates nonalcoholic fatty liver disease via modulating the gut microbiota
Source: Microbiol Spectr. 2024 Apr 22;12(6):e01979-23. doi: 10.1128/spectrum.01979-23 (PMC11237417; doi:10.1128/spectrum.01979-23)
Supplement: Table S1 — Primer sequences for RT-PCR. [file spectrum.01979-23-s0006.pdf]

Supplementary Table 1 The primer sequences for RT-PCR

**Gene Symbol**	**Forward Primer**	**Reverse Primer**
GAPDH	GTGTTCCTACCCCCAATGTGT	ATTGTCATACCAGGAAATGAGCTT
Scd1	TCCTCCTTGGATTGTGTAGAAACTT	AATGTCAGAAGAAATCAGGTGGGTA
CD36	ATGGGCTGTGATCGGAACTG	GTCTTCCCAATAAGCATGTCTCC
fas	ATCGCCTATGGTTGTTG	TCACGACTGGAGGTTCTA
PPARγ	GAAAGACAACGGACAAATCACCAT	CGGCTTCTACGGATCGAAACTG
Adrb3	CCTTGGGCGAAACTGGTTG	GTTGGTGACAGCTAGGTAGCG
Lipe	TGGCACACCATTTTGACCTG	TTGCGGTTAGAAGCCACATAG
Pnpla2	GGATGGCGGCATTTCAGACA	CAAAGGGTTGGGTTGGTTCAG
Cpt2	CAAAAGACTCATCCGCTTTGTTC	CATCACGACTGGGTTTGGGTA
Acox1	TAACTTCCTCACTCGAAGCCA	AGTTCCATGACCCATCTCTGTC
Ppargc1α	TATGGAGTGACATAGAGTGTGCT	CCACTTCAATCCACCCAGAAAG
PPARα	TACTGCCGTTTTCACAAGTGC	AGGTCGTGTTCACAGGTAAGA
Fabp5	GGAAGGAGAGCACGATAACAAGA	GGTGGCATTGTTCATGACACA
Occludin	GTGGTTTGACACTGACTTCCC	CTCCTCTCGGTGACAGAGTCT
ZO-1	TTTTTGACAGGGGGAGTGG	TGCTGCAGAGGTCAAAGTTCAAG
Muc5	GTGGTTTGACACTGACTTCCC	CTCCTCTCGGTGACAGAGTCT
CD14	GAGTTGTGACTGGCCCAGTCAGC	GCAAAAGCCAGAGTTCCTGAC
TLR2	AAGATGCGCTTCCTGAATTTG	TCCAGCGTCTGAGGAATGC
TLR4	TGTTCTTCTCCTGCCTGACA	CATCAGGGACTTTGCTGAGTT
NLRC4	CGGCCTGCAACCTCTTTCTT	TGGGCCAAAACATTCAGGTCT
MCP-1	ATCCCAATGAGTAGGCTGGAGAGC	CAGAAGTGCTTGAGGTGGTTGTG
IL-10	TGTCAAATTCATTCATGGCCT	ATCGATTTCTCCCCTGTGAA
CYP7A1	AGCAACTAAACAACCTGCCAGTACTA	GTCCGGATATTCAAGGATGCA
CYP27A1	GCCTTGCACAAGGAAGTGACT	CGCAGGGTCTCCTTAATCACA
FgFr4	GATGGACAGGCCTTCCACGGG	GGTTGCTGTTGTCCACGTGAGGTCTTC
FXR	TGTGAGGGCTGCAAAGGTT	ACATCCCCATCTTGGAC
shp	TCTGGAGCCTTGAGCTGGGT	GCCTTGGCTGGCTGGGTAC
Fgf15	CCAACTGCTTCCTCCGAATCC	TACAGTCTTCCTCCGAGTAGC
SLC5A8	TCGAGTTGGCGAAGGGGACCA	ATGCCTTGGCGGCAGTCACC
Srebp1c	GAGCGAGCGTTGAACTGTAT	ATGCTGGAGCTGACAGAGAA
